# An Atypical Presentation of Lichen Planus-Like Reaction from Pembrolizumab

**DOI:** 10.1155/2019/4065437

**Published:** 2019-07-07

**Authors:** Matthew Lee, Nagashree Seetharamu

**Affiliations:** ^1^Department of Medicine, North Shore University Hospital-Long Island Jewish Medical Center, 300 Community Dr, Manhasset, NY 11030, USA; ^2^Department of Oncology, Donald and Barbara Zucker School of Medicine at Hofstra/Northwell, 500 Hofstra Blvd, Hempstead, NY 11549, USA

## Abstract

**Introduction:**

With the advent of immunotherapy, a new subtype of side effects called IRAEs or immune related adverse effects have become more common. They may present in various organ systems as colitis, pneumonitis, hypophysitis, and thyroiditis and commonly as dermatological reactions.

**Case:**

This is a case report of a lung cancer patient that was started on Pembrolizumab and developed shortly after what appeared to be clinically at first pustular psoriasis but on biopsy was confirmed to be lichen planus. She was discontinued on the Pembrolizumab and treated with both systemic and topical steroids and improved.

**Conclusion:**

This case highlights a cutaneous reaction from Pembrolizumab and the subsequent management that helped resolve her condition but also weighing the benefits against the risk of treatments and potential prognostic implications of having cutaneous side effects.

## 1. Introduction

Cancer growth has long been linked to the ability of malignant cells to avoid detection by the patient's own immune system and leads to uninhibited growth. With increased understanding of tumor immunity, new medications have been developed to selectively target checkpoints in the cell cycle known as checkpoint inhibitors. Some of the most common targets include programed cell death protein and ligand-1 (PD-1/PD-L1) and cytotoxic T lymphocyte associated protein-4 (CTLA-4) now utilized in metastatic melanoma, lung cancer, and renal, gastric, and hepatocellular carcinomas management [1, 2]. Activating and improving the patient's own innate immune system to recognize and to act against cancer cells have led to improved outcomes with less toxicity than traditional chemotherapy. However, this has also led to nonspecific activation that causes a new class of side effects known as immune-related adverse side effects (IRAEs). Examples of the most common side effects related to checkpoint inhibitors are colitis, diarrhea, thyroiditis, and hypothyroidism with autoantibodies against thyroid peroxidase or thyroid stimulating hormone receptors, hypophysitis, pneumonitis, autoimmune hepatitis, and polyarthralgia. Of these IRAEs, dermatological reactions are the most common including pruritus, maculopapular rashes, vitiligo, lichenoid skin reactions, psoriasis, and rarely life threatening effects like bullous pemphigoid, Stevens-Johnson syndrome, and drug rash with eosinophilia and systemic symptoms (DRESS) [3, 4]. In this case report, the patient was on Pembrolizumab and developed lichen planus and we will discuss further its presentation, management, and potential prognostic implications if patients have cutaneous side effects from immunotherapy.

## 2. Case

**Figure 1:**
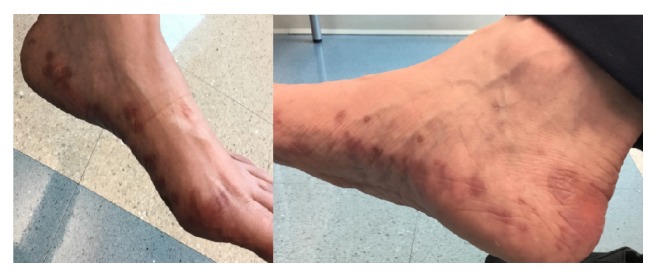
Photograph of lichen planus lesions on the patient's feet.

**Figure 2:**
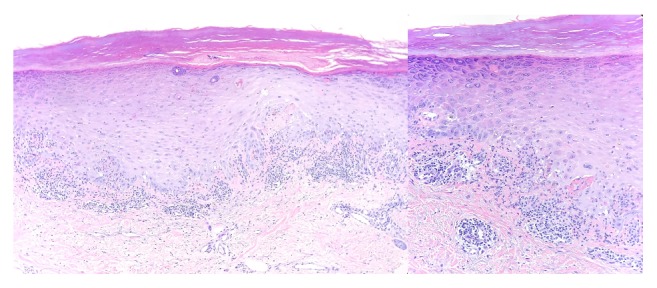
Skin biopsy shows a lichenoid lymphocytic infiltrate with acanthosis, hyperkeratosis, hypergranulosis, and civatte bodies (apoptotic basal keratinocytes).

This is a case of a 60-year-old female with stage IV T3N3M1 lung adenocarcinoma metastatic to adrenals and bone was found to have EGF, ROS, ALK WT, and KRAS mutation and lymphangitic disease. She initially received six cycles of carboplatin and pemetrexed and then switched to maintenance Alimta after three cycles. However, CT scans showed progression of disease and she was switched to Nivolumab. For dosing convenience for the patient, she was then switched to Pembrolizumab for every 3 weeks. Five months since starting Pembrolizumab, the patient then developed a pruritic rash on her wrists, feet, and buttocks and oral mucosal lesions. She was given clobetasol ointment and benadryl but on a follow-up visit a month afterwards she had developed a worsening rash. It became more difficult to walk and on physical exam the lesions appeared to be fluid filled and weeping bilaterally on feet and hands with psoriasiform papules, patches on back and buttocks, indurated pustules, and plaques on palms and sole. It was initially diagnosed by her dermatologist on clinical exam to be possibly pustular psoriasis and the patient was then started on oral prednisone 20 mg once a day and urea cream 40% twice a day and her Pembrolizumab was held. She improved within the next week but still had persistent maculopapular lesions on her feet (Figure 1) and then a punch biopsy of a left thigh lesion showed a diagnosis of palmoplantar lichen planus reaction (Figure 2). After five months of stopping the Pembrolizumab the rash started to decrease and her oral mucosal lesions disappeared. One year after the first cycle of Pembrolizumab, the patient now has healing hyperpigmented lesions with residual pain where the prior lesions were with Nystatin-Triamcinolone 10000-0.1 unit/gm external cream three times a day and Pembrolizumab is still being held. Furthermore, her lung adenocarcinoma is currently well maintained and has not progressed despite having Pembrolizumab being held and no further treatment.

## 3. Discussion

Although there is a wide variety of IRAEs, cutaneous side effects are the most commonly associated with checkpoint inhibitors. In a study by Hofman et al. it has been reported that 8.7% of melanoma patients treated with anti-PD1 therapy developed primary adverse dermatological events [5]. Specifically, Pembrolizumab, an IgG4 antagonist antibody to PD-1, was associated with cutaneous adverse events in 42% of its patients and 8 months from onset on average [6–9].

Lichen planus and lichenoid reactions are chronic inflammatory, T cell mediated reactions to an unknown antigen seen in various medications including beta-blockers, antimalarials, antihypertensive and proton pump inhibitors. Clinically, lichenoid reactions have different subtypes based on the sites it is involved with and morphology. Classically, it presents as papular/plaques, purple, pruritic, polygonal, and planar. Other types include hypertrophic that is common on extremities, vesiculobullous with blisters on lower extremities, and palmoplantar lesions that are scaly, bilateral, and symmetrical on malleoli and internal plantar arch. Other locations lichen planus can affect other than cutaneous locations are mucous membranes including oral, vulvovaginal, conjunctival, and laryngeal/esophageal [10–15]. As in our case, the patient presented with bullous vesicular lesions originally thought to be pustular psoriasis due to the clinical picture but found on biopsy to be ultimately lichen planus. Moreover, the lesions were also found on her oral mucosa along with flat papular polygonal lesions bilaterally on the plantar arch and malleoli areas.

The mechanism of how this occurs is still unknown but is thought to be T cell mediated. PD-1 itself is an inhibitory molecule on T cells that have immune tolerance to self-antigens and malignant tumors can express PD-L1 in order to evade immune responses [10]. PD-1 is also involved in epidermal preservation during inflammatory reactions and, by blocking PD-L1, there is not only an increase in the immune function of tumor-specific T cells but also an unmasking effect of self-immunity or prior antigen immune response. This leads to a widespread nonspecific T cell activation with an increase in T cell and TCR binding resulting in IRAEs in multiple organ systems and cutaneous reactions. In lichenoid reactions, they specifically affect keratinocytes expressing PD-L1 with lymphocyte infiltration in subepithelium and necrosis of keratinocytes, dense CD4 positive and CD8 positive T cells, spongiotic dermatitis, acanthosis, lymphocytic infiltrate of basal membrane, and hypergranulosis [10–15]. Interestingly, lichenoid reactions are not seen in other targeted therapies such as Ipilimumab, Epidermal Growth Factor Receptor (EGFR) inhibitors like Erlotinib and Bevacizumab or chemotherapies [9]. This may indicate that cutaneous reactions are a target effect on PD-1/PD-L1 pathway than nonspecific hypersensitivity reaction.

Treatment for cutaneous IRAEs in general is based primarily on grade severity which is referred most commonly by The National Cancer Institute's Common Terminology Criteria for Adverse Events (CTCAE) [16] (Table 1). However, psoriasis and lichenoid lesions were not specifically mentioned in CTCAE version 5.0 [16]. Furthermore, there is no standardized treatment for many IRAEs and management is based on case reports, case series, and expert consensus and opinions. Generally, Grade 1 and 2 cutaneous reactions and events are treated with topical corticosteroids and an oral antipruritic or antihistamine. For Grade 3-4 a skin biopsy is needed for classification and systemic steroids are needed for at least 2-4 weeks. Steroids can be gradually reduced over 1 month if there is a response with most IRAEs resolving within 6-12 weeks [17]. Furthermore, cessation of immunotherapy (temporary or permanent) is needed with these high-grade reactions [5, 18, 19]. Lastly is the case of being refractory to steroids, immunomodulatory or immunosuppressive agents such as TNF-alpha (tumour necrosis factor) antagonists, azathioprine, and MMF (mycophenolate mofetil) [20, 21].

In terms of specifically for lichenoid reactions the main management includes topical and systemic corticosteroids [22]. The majority are able to be maintained on their anti-PD1 therapy (81%) and it was concluded that treatment can still be continued [23]. Furthermore, it has been reported that the majority respond well to topical steroids and holding immunotherapy may not be needed in contrast to more severe immunobullous toxicities [24]. Evidence of reduced efficacy of immunotherapy with immunosuppressive medications is mixed and no definitive conclusions [25, 26] and it is preferred still especially in high-grade cutaneous toxicity as it can enable reinitiation of immunotherapy [25]. In this atypical case of lichen planus, the lesions were initially severe enough to cause our patient substantial clinical effects and concerning features of bullous lesions that were resistant to topical steroids that oral steroids were needed and cessation of therapy in order for it to finally resolve.

Many of the cutaneous lichenoid side effects resolve within 6 months to 1 year. However, it can also reoccur and have periods of waxing and waning depending on different subtypes including erosive lichen planus or hypertrophic variants [27]. In terms of prognosis, cutaneous side effects have also been shown as potential positive prognostic factors. A study by Sanlorenzo et al. had examined a sample of patients treated with Pembrolizumab who had cutaneous adverse effects and their survival analysis showed that those who had cutaneous adverse effects had longer progression free intervals irregardless of treatment regimens [6]. Another study by Freeman et al. involved Nivolumab and metastatic melanoma patients and also showing a longer progression free survival compared to those who did not experience cutaneous toxicity [28]. Furthermore, adverse cutaneous effects such as hypopigmentation and vitiligo have been shown to be possible positive prognostic factors but mainly in melanoma patients [29–31]. However, what could potentially affect these findings is that patients who progress with immunotherapies like pembrolizumab or nivolumab would not have the same cumulative amount compared to those who do not progress and continue taking it. Thus, those that stay on treatment have a higher chance of developing a cutaneous adverse effect and may have a longer progression-free survival.

Lastly, there is controversy over whether oral lichen planus is a potential risk factor for oral squamous cell carcinoma (SCC). It has been debated that since oral lichen planus has been shown to have had a loss of heterozygosity and microsatellite instability and possible risk of malignant transformation [32]. Further, it was shown that 2.4% of oral lichen planus patients developed oral SCC in previously treated areas [33] with transformation ratios ranging from 1% to 5% [34, 35]. However, there is a need to have larger prospective cohort studies and further studies.

## 4. Conclusion

As for this patient in this case report she had developed a lichen planus eruption after 3 months of an anti-PD-L1 treatment, Pembrolizumab. Management for her case included topical treatment that initially failed and then systemic treatment with steroids and cessation of Pembrolizumab which eventually led to the resolution of the rashes and cutaneous lesions. This case highlights how different ways cutaneous side effects can be managed and present as and the subsequent difficulties based on clinical exam alone. Recognizing toxicities from immune checkpoint therapies becomes increasingly important as the number of patients on these treatments continues to grow and decisions on whether to stop or continue immunotherapies continue to be based on clinical experience as the field advances. Side effects such as vitiligo and hypopigmentation have been shown to be potentially prognostic and future studies should examine if other cutaneous findings can be also.

## Figures and Tables

**Table 1:** Skin and subcutaneous tissue disorders management of different gradings based on CTCAE (Common Terminology Criteria for Adverse Events) [16].

Grading (CTCAE)	General Cutaneous Features	Steroids	Immunosuppressives	Management of Immunotherapy	Outpatient or Inpatient care
1	Covers <10% BSA, limited, asymptomatic	Not recommended	Not recommended	Continue	Outpatient

2	Covers 10-30% BSA, minimal to moderate symptoms	Topical or systemic steroids oral 0.5-1 mg/kg/d	Not recommended	Continue	Outpatient

3	Covers >30% BSA, moderate or severe symptoms, limiting self-care ADLs	Systemic steroids oral or IV 1-2 mg/kg/d for 3 days then to 1mg/kg/d	Can consider if unresolved after 3-5 days of steroids	Suspend, discuss risk/benefit with patient	Outpatient or Inpatient for IV steroids

4	>30% BSA and fluid/electrolyte abnormalities	Systemic IV methylprednisolone 1-2mg/kg/d for 3 days	Can consider if unresolved after 3-5 days of steroids	Discontinue permanently	Inpatient and possible ICU or burn unit

BSA: body surface area.

## Data Availability

The datasets supporting the conclusions of this article are included within the article.

## Abbreviations

PD-1/PD-L1: Programed cell death protein-1 and programed cell death ligand-1
CTLA-4: Cytotoxic T lymphocyte associated protein-4
IRAEs: Immune-related adverse side effects
TSH: Thyroid stimulating hormone
DRESS: Drug rash with eosinophilia and systemic symptoms
EGF: Epidermal growth factor
ROS: Protooncogene tyrosine protein kinase
ALK WT: Anaplastic Lymphoma Kinase
KRAS: Oncogene Kristen Rat Sarcoma
CTCAE: Common Terminology Criteria for Adverse Events
AE: Adverse events.
